# Citizen science characterization of meanings of toponyms of Kenya: a shared heritage

**DOI:** 10.1007/s10708-022-10640-5

**Published:** 2022-04-16

**Authors:** Nyangweso Daniel, Gede Mátyás

**Affiliations:** 1 PhD School of Earth Sciences, ELTE Eötvös Loránd University, Pázmány Péter sétány 1/A, H-1117 Budapest, Hungary; 2 Institute of Cartography and Geoinformatics, ELTE Eötvös Loránd University, Pázmany Péter sétány 1/A, H-1117 Budapest, Hungary

**Keywords:** AGI, Heritage, VGI, Gazetteer, Toponyms

## Abstract

This paper examines the toponymic heritage used in Kenya’s Authoritative Geographic Information (AGI) toponyms database of 26,600 gazetteer records through documentation and characterization of meanings of place names in topographic mapping. A comparison was carried out between AGI and GeoNames and between AGI and OpenStreetMap (OSM) volunteered records. A total of 15,000 toponymic matchings were found. Out of these, 1567 toponyms were then extracted for further scrutiny using AGI data in the historical records and from respondents on toponyms’ meanings. Experts in toponymy assisted in verifying these data. From the questionnaire responses, 235 names occurred in more than one place while AGI data had 284. The elements used to characterize the toponyms included historical perceptions of heritage evident in toponyms in their localities, ethnographic, toponymical and morphology studies on Kenya's dialects. There was no significant relationship established between the same place name usages among dialects as indicated by a positive weak correlation *r* (438), = 0.166, *p* < 0.001 based on the effect of using the related places and the distance between related places. The weak correlation implies that the one name one place principle does not apply due to diverse language boundaries, strong bonds associated with historical toponyms in the form of heritage and significant variations on how names resist changes to preserve their heritage.

## Introduction

Toponyms are geographical names or names of places especially those associated with topographical features. Etymological derivations of toponyms are inspired by natural phenomena (Tent, 2015), thus providing clues for analyzing the toponyms’ linguistic and cultural history. Generally, a higher percentage of the place names in Kenya is named after prominent topographic features or environmental conditions. The chronological evidence approach which traces the origin and date for each toponym in Kenya is scanty due to the non-documentation of most records. Kenya's lingua franca is the Swahili orthography, which is used for all geographical names designating places and topographical features. The use of Swahili does not offer translation of the place names with diacritical marks or symbols for languages in Kenya. Thus, the toponyms’ specific vernacular spellings are different from the original written script of pronunciation such as those of England (Moriarty, 2021; Sharpe, 2011). Some connect to sources such as Romans, Germans and Celts as documented by Moriarty’s online article. Other factors affecting place-name heritage are non-documentation, usage changes, changing names and informal place names other than the officially sanctioned ones for all related dialects. However, whether documented or not, each place name has historical sources (Bühnen, 1992).

Bühnen further posits that toponyms documentation started as early as the ninth century for the Arabic records on Sub-Saharan Africa (strictly for the Sahel and North Africa). This documentation excluded most parts of East Africa. In the European region, it began in the fifteenth century, where by the nineteenth century, significant documentations were published (Hausner, 2017). For example, when parts of East Africa were unmapped, the source of river Nile then was considered as a fictional “mountain of the moon” instead of Lake Victoria. The confusion about the source of the Nile shows how there was little documentation on most parts of East Africa until the nineteenth century. Studies of place names by scholars are sometimes neither documented nor shared with the National Mapping Agencies (NMA) for publishing in topographic maps leading to gaps in non-documented toponymy.

This paper assesses Kenya's 1567 toponyms’ connotated meanings based on available features and the official gazetteer containing 26,600 toponym records. 15,000 records were found both in Volunteered Geographic Information (VGI) and Authoritative Geographic Information (AGI) records. Out of these 15,000 records, only 1567 records were used in characterizing heritage. Toponyms that were not included in heritage documentation are street names, nicknames and mistake names since they do not carry heritage and often suffer from correct lexicon or effect of geopolitics unlike toponyms from other themes. It was easier to compile historical records since there is no significant effect on the toponyms in retrieving actual characteristics and translated or implied meaning due to Swahili orthography usage for all the 45 different lexicons or languages.

### Characterization of toponym typologies and origins

Various typologies used in studying toponym origin are proposals from names authorities, toponymy scholars and the United Nations Group of Experts on Geographical Names (UNGEGN). The type of typology to use depends on the mapping institution's guidelines or researchers’ aim and the mapped features. Typology treatises from researchers and technical guidelines from mapping institutions are taken as core guidelines for use within a given governing entity such as the Australian National Place Names Survey (ANPS). The ANPS has a series of Technical papers or working paper for classifying toponymic origins. The UNGEGN technical papers indicate that the toponymy manual exist emanating from the 1959 and 1967 resolutions about social and cultural values of toponyms (Helleland, 2002) in addition to individual scholarly article contributions. Place name origin has been a widely researched topic in recent years by various scholars (Bölling, 2013; Jenkins, 2018; Klugah, 2013; Laaboudi & Marouane, 2018; Stewart, 1954; Tent & Blair, 2011). In these studies, the most used typology includes descriptive, occurrent, associative, evaluative, shift, indigenous, eponymous, linguistic, innovation and erroneous dimensions. Most countries have developed their methodologies of a typology to adopt. For example, Australia, through its Place Name Survey (ANPS) office, proposed a toponym typology that contains descriptive, associative, occurrent (events), evaluative, copied (shift), eponymous and innovative names for use nationally. However, the recently published Paper No. 5 typology agrees with most of Stewart’s classification though there are differences in feature landscapes’ heterogeneity and the use of complex subgroups within Australia’s proposed typology.

Researchers of exonyms in South African toponymy involving the influence of the Portuguese language found that names of seafarers and dignitaries, saints and kings, hydronyms, cattle and herders, marine animals and features of vegetation topography were used as classes for analysis. The context of the study referred to modified geographical names (Meiring, 2008). An investigation of various toponyms classification on naming motivations in multiple countries (Tent & Blair, 2011) posits that the giver of toponyms and those assigning categories appear to disagree due to the non-documentation of namer’s intentions or motivations. The situation gets complicated when different locations apply since there is a lack of an ideal toponym typology standard (Tent, 2015; Tent & Blair, 2011) suited for all areas in each country. However, Stewart’s classification of 1954 (later improved in 1975) provides a basis for any toponymist to make a meaningful classification of toponym typology suited for any area of interest.

There has been an application of statistical research of toponyms in studies such as Bray–Curtis, Euleadian and Cambera which evaluated dialects’ onomageographical boundaries (Ditrói, 2018). The Bray–Curtis methodology offered better results though with some uncertainties on the algorithm. There is also the application of the spatial smoothing method (Qian et al., 2016) in analyzing the distribution of ethnic toponyms whose aim was to get ratio distributions. However, research on statistical toponymic heritage mapping of insights of namers’ intentions or motivations is scanty especially those evaluating social usage such as toponyms collected from questionnaires. Different ways of obtaining toponyms data include making field visits, personal interviews, ethnographic surveys, focus group discussions and questionnaire surveys. The use of the methods and the challenges that may arise lies in the documentation and easy access or reference; a case which has made most National Mapping Agencies (NMA) seek volunteer contributed data for enriching gazetteer data (Gao et al., 2017; Keßler et al., 2009; Oliveira et al., 2016). Currently, mobile applications help in collecting data on toponym heritage (Baglioni et al., 2007; Robinson et al., 2017) in addition to classical methods.

Geographical names are changing not only on location, application, recognition and use but also on documentation and presentation on different platforms. The changes in geographical names seem to be due to toponyms that are not familiar to the locals or to place names that have little historical significance (Perdana & Ostermann, 2018). However, for toponyms continuously used, changing them is hard and if changed, their adoption takes time before replaced toponyms become historical names.

### Toponyms as shared cultural heritage

Toponyms carry a symbol of intangible cultural heritage and are shareable within a group of dialects in a region, country or across borders and can reveal culture and language contact (Möller, 2019) as indicated by the study on the Bantu, Bushmen, Khoikhoi and Dutch of South Africa interaction. The role of toponym heritage in society ranges from the reflection of local and national identity, protection of intangible social rights held by groups of people in the preservation of culture (Jordan et al., 2009; Lauder, 2013) to acting as reference objects for the tourism sector (Lemmi & Tangheroni, 2011; Lim & Cacciafoco, 2020).

There is an ongoing debate on the correct definition of constructing and defining a shared heritage. However, there are varieties of cultural heritage definitions coming from organizations of heritage such as United Nations Educational, Scientific and Cultural Organization (UNESCO), International Council on Monuments and Sites (ICOMOS) to scholarly documents (Ahmad, 2006; Petti et al., 2019). This paper focuses on shared toponym cultural heritage within dialects and how they are used as symbols of intangible heritage (Kerfoot & Cantile, 2016) in Kenya. The research assessed toponyms heritage as reflected in multiple usages in Kenya through web maps. The objective was to find out the best approaches in updating data in the public and VGI sourced information platforms through development and service rendering. Same place-name usages across different dialects reflect in their application in historical and newly collected data from respondents in areas outside native boundaries.

This paper discusses VGI toponyms data collection techniques and approaches by considering aspects of toponym heritage and characterization from the user’s perspective using test cases of questionnaire responses by using an app and a website to submit VGI data. It is organized in six sections. The first section introduces toponyms concepts such as geographical names of places and its context in characterizing heritage of VGI data. It further explores characterization of toponym typologies and motivation of the shared heritage. The second section makes a review on toponym characterization. The third section covers the methodology used in the application of VGI as a method in collecting and compiling toponym heritage information from citizens using the web and mobile techniques by use of questionnaire response data. Besides, it provides insights into how information perceptions existing in the local domain about different toponyms can be collected and related to each other using features and places in a web map. Various data sources were explored to lay out the case on contributions made by citizens in submitting VGI data based on the information contributed ranging from related-places data and provision of heritage information on toponyms. Respondents also captured the heritage information by using either a pre-developed mobile app and an online questionnaire or using web-map links to collect and visualize toponyms as captured in real-time. The efficacy test of characterization of toponyms was done by asking respondents to provide feedback on their experiences in collecting heritage information for statistical analysis to further improve the methodology. The fourth section provides results arising after subjecting statistical analysis to the VGI data contributed by citizens in the study area. The fifth section gives a discussion regarding the respondents’ description information to provide distinctive meanings and their relationship with others located elsewhere. Finally, the sixth section outlines how place-name representations scribe spatial heritage of resilience, memory of space, character and physical terrain. This is supported by concurrent views of information regarding the historical heritage information on Kenya's geographical names from different dialects as indicated by sampled questionnaire data.

## Literature review

Citizen science generally involves the community or individual members of the public voluntarily taking part in a research project in order to fill authoritative data gaps by providing VGI data. The contributors help to make discoveries in research using applications and technologies for purposes of solving human and environmental problems though public participation. Scholarly interpretation of citizen science takes different forms based on the degree of participation (Heigl et al., 2019). Citizen science approach has been in use in various institutions such as the World Bank’s Global Facility for Disaster Reduction and Recovery (GFDRR), Fintan Project in Sudan (Haklay et al., 2014), Swedish Lantmäteriet project^1^Gävle city, Fix my Street (Walravens, 2013) and Map Gretel (Rönneberg et al., 2018) where each project had different goals and approaches. In VGI data collection, standard procedures are needed for better data quality results and improved usability (Felgenhauer, 2018; GFDRR & World Bank, 2018; Kostanski et al., 2012; Ormeling, 2017). When retrieving indexed entries from different VGI data sources, it is better to compare official data in order to mitigate its shortcomings in content, coverage and quality (Mahabir et al., 2017). Existing guidelines for successful VGI application may have logistical challenges in VGI data compilation since data is generated from different people with varying diverse skills, which NMA’s must overcome to realize the full potential of VGI. Besides, attribute schema for gazetteer data collection procedures varies across various government agencies. There is an increased usage of the Global Positioning System (GPS), open-source systems and internet-enabled devices with enhanced VGI data collection (Rice et al., 2012). Although there is an increased use of mobile applications most of which aim at direction finding between points of interest (Freundschuh, 1989, 1991; Gopal & Smith, 1990; Gould, 1989; Mark, 1989) they do not support attributes of heritage and relations as part of data collected.

### Characterizations of toponyms origins

Studies on toponyms characterization exist, such as in Italy where an investigation of toponyms in the Ogliastra sub-region of Sardinia focused on linking soil and land perception with knowledge (Capra et al., 2015). However, Capra’s typology does not classify mixed dialects, clans, personal names and their contribution in naming as per historical events. In a different study in Australia, a review of indigenous animals established a strong link between names given to the animals and the indigenous people (Peter, 2017). Some scholars have also employed Natural Language Processing (NLP) systems in toponym investigation (Barbaresi, 2018; Wolf et al., 2014) for visualization. Results indicated that NLP systems are figuratively used during indexing and querying unless they are interpreted first before indexing and querying. In Finland, a study on Finnish toponyms (Kaups, 1966) used typologies such as descriptive, personal, ethnic, commemorative, others and abandoned. Kaup’s study considered physical features like creeks, bays, lakes and others as classes. In contrast, cultural features category included hamlets, townships and others but left toponyms emanating from topographic nature such as soil colour, the presence of rocks, or vegetation. Though classifications of toponyms are open, having many themes may have merits and demerits (Urazmetova & Shamsutdinova, 2017) unlike when having few categories, hence posing challenges in data entry in content and quality. A comprehensive list of toponyms and origins was done for some toponyms in the United States (Gannett, 1905) and involved significant personnel and time as documented. Even though there is no typology classification for the United States toponyms, toponyms were all arranged in a sequential alphabetical list with meanings. Besides, a vocabulary of place names developed for South Africa (Möller, 2019) closely follows the approach of the United States.

In Kenya, obtaining information on onomastic publications is not easy. In most cases, the studies target a specific area involving one area or dialect group hence, one cannot categorize the toponyms to make relative perception and relations in toponyms wholesomely. Typical examples include a study concentrating only on three informal settlements (Wanjiru & Matsubara, 2017) and anchored how toponyms represent the urban landscape. A morpho semantics study done for Luhyia’s Lulogooni sub-language covered 64 toponyms (Anindo, 2016). The study covered a small area that did not include attributes like changes, toponyms coordinates and the relations of those toponyms with other dialects in Kenya. Similarly, another study on the Kipsigis dialect (Kibet & Mwangi, 2016) considered 56 toponyms based on Anindo’s approach. In both cases of Lulogooni and Kipsigis studies, the toponyms covered were few. Their areas may have spatial heterogeneity differences if a different location with a different dialect applies due to the possibility of getting new features.

Toponyms serve as a descriptive text describing gazetteers (Goodchild & Hill, 2008; Keßler et al., 2009). However, there is no dialect information available in VGI records of OpenStreetMap (OSM) and GeoNames data to aid toponymy studies such as factors influencing naming using VGI, such as culture, language and tribal group identities (Alasli, 2019; Morgan, 2000). Early civilizations across the world used totem symbols of animals and their body parts or plants as identities for groups of people (Frazer, 1910; Kigen & Hans, 2018) when the boundaries were clear. The purpose of totems is to offer identification and associations within dialects, clans and cultures with specific places in their diverse heterogeneous environment of heritage centred on beliefs.. However, the totems are only distinct to a specific sub-dialect within a dialect such as the studies on the Dorobo (Huntingford, 1929) and dialects in most parts of the globe (Frazer, 1910). Due to urbanization and geopolitics, ethnicity can determine and motivate a populace to model desired voting patterns rather than reference sites. A problem may arise from minority dialects such as Yaaku, Dahalo, Dassenach, Ogiek, El Molo, Waata, Aweeri, and Sanye, Boni (Makoloo, 2005) among others whose identities miss in naming places where the main dialect reside beside them. In this context, dialects may be in existence but with no identical recognitions. In a coexisted society, place names, if used, protect the minorities in their representation in planning and development through naming regions inhabited by minorities without strictly considering the standard electoral rules. Typical examples of these are the Maasai toponyms used entirely almost in Kenya’s major towns in the Rift Valley region, the use of minority place names in some places not inhabited by any of the minorities especially in streets and estates of towns. Although Kenya’s toponyms use Swahili orthography, the actual *Swahili* and *Shirazi* place names appear concentrated only on the coastal towns where almost all toponyms have a Swahili dialect affiliation.

Toponyms are aspects which humans relate with everywhere at all levels. Their application is in administration, sustainable economic and planning policies, environmental management, emergency response coordination, trade, cultural heritage, utility and facility infrastructure management, tourism, intelligence communication systems and the media (UNGEGN, 2000). The 9th UNGEGN conference of 2007 regarding resolution 2007-res 7(ix/7)(UNGEGN, 2007) proposed the use of toponyms’ origin and meanings in gazetteer content which is yet to be done by most countries. The concept of tracing the importance of place names reveals a culture’s roots (Taylor, 1966), settlement patterns (Senekal, 2019), events and cultural documentation. To date, most countries have launched web gazetteers with free access but there is no inclusion of sources of the named places as part of the gazetteer content of the place names. To assess the heritage and the associated characterized meaning of each place name, first records were collected from the existing vocabulary of toponyms and origins based on existing recognized dialects in Kenya as indicated in Table 1.

**Table 1:** Toponym characterization based on dialects (number of toponyms = 1,67, dialects 26)

	Dialect name	No. of toponyms	% toponyms		Dialect name	No. of toponyms	% toponyms	
1	Kalenjin	234	14.9%	16	Orma	23	1.5%	
2	Kikuyu	200	12.8%	17	Turkana	15	1.0%	
3	Maasai	186	11.9%	18	Taita	9	0.6%	
4	Luhyia	132	8.4%	19	Pokomo	8	0.5%	
5	Mijikenda	123	7.8%	20	Meru	5	0.3%	
6	Kamba	109	7.0%	21	Arab	2	0.1%	
7	Samburu	91	5.8%	22	Teso	1	0.1%	
8	Swahili	73	4.7%	23	Tharaka	1	0.1%	
9	Luo	72	4.6%	24	Sakuye	1	0.1%	
10	Somali	60	3.8%	25	Taveta	1	0.1%	
11	Boran	52	3.3%	26	Nubi	1	0.1%	
12	Kisii	48	3.1%	27	Others*	0	0.0%	
13	English	48	3.1%					
14	Embu	43	2.7%					
15	Gabbra	29	1.9%					
			Total			1567	100%	

As shown in Table 1, some dialects represented by the “other” group lacked toponyms for characterization since there were no authentic historical documents for those dialects. Table 1 shows that formal documentation of place names is incomplete and needs to be updated on a regular basis. Table 1 shows that the results collected from documentation based on formal card records do not adequately represent all of the 42 dialects that existed in 1962 due to a lack of regular documentation to update the records by the NMA.  

### Toponyms studies in Kenya

Toponym research has been ongoing worldwide and locally (Peng et al., 2019; Reinsma, 2017; Tent, 2015). About 45 dialects reside in Kenya (KNBS, 2019). This research is examining the etymology, meaning and origin of toponyms. Studies carried out on toponyms focused on totems and ethnographic studies involving groups of languages to individual sub-ethnic communities (Frazer, 1910; Hobley, 1898; Kiriama, 1986; Ndeda, 2019). The studies range from ethnographic studies to field visits as per the documented historical data on dialects, totems and clans in Kenya. Other studies include those for the dialects such as the Luhyia (Anindo, 2016),the Turkana (Lamphear, 1998) and all the Bantu dialects(Van de Velde, Nurse and Bostoen, 2006). There is also the historical study for the Meru (Icheria, 2015), the Taveta (Momanyi, 2002), the Masai (Little, 1998; Mwangi et al., 2006; Richmond, 2016; Seno & Tome, 2013), the Kisii (Omwenga et al., 2015; Omwoyo & Mildred, 2000), the Kipsigis (Kibet & Mwangi, 2016) and the Kikuyu (Kenyatta, 1938). Minority dialects contain information about the lost people, the Gumba (Kenyatta, 1938; Taylor, 1966). As of 1962, there were about forty known dialects or tribes (Morgan, 2000) that contributed names for the toponyms. Currently, five more dialects have recognition as per the new Constitution (The National Council for Law Reporting, 2010). These dialects emanate from the minority groups such as the Dorobo, Makonde, Asians, Sakuye and Dasenach.

## Data and methodology

Examination of the meanings and characterization of toponymic heritage considered Kenya’s AGI toponyms database of 26,600 gazetteer records as of the year 1959 based on seven classes on the sources of the toponyms as shown in Table 2. A comparison of GeoNames and OSM’s volunteered records was made with AGI’s 26,600 gazetteer records and found 15,000 toponym matching. 17,567 records for GeoNames and 15,000 for OSM matched those records contained in AGI. 9334 records for GeoNames and 11,600 for OSM did not match probably due to name changes through decolonization, erased or shifts from the original data or the features identified then no longer existed. In addition to historical manuscripts, research articles and an online questionnaire, 1567 toponyms were sampled from the original set and were analysed. Historical and online questionnaires were compiled, authenticated and validated by toponymic experts.

**Table 2:** Toponym characterization based on features in the AGI database of toponyms (*n* = 26,600)

Class	Category of toponyms sources	% contribution
1	-Topographic nature of the area -Soil type, texture and color, land cover types, e.g., plants, -Vegetation, trees and shrubs -Watercolor, size and nature	57%
2	Personal names, tribes, sub-tribes and clans	15%
3	Animals, birds, insects and worms	7%
4	Historical events, rituals, sacrifices, religion with gods and God	12%
5	Disasters and natural calamities like diseases, earthquakes	1%
6	Household goods	5%
7	Foreign names and opaque toponyms	3%
	Total	100%

The approach on this study used seven classes of derived meanings for characterization based on available data, after a review of (Stewart, 1954) and (Tent, 2015) approaches of classifying toponym-origins into seven categories shown in Table 2.Topographical features such as soil, vegetation and hydrological features formed the first category. This category represented 57% of the 26,600 gazetteer records. The second category included toponyms of Kenyan personal names, tribes, sub-tribes and clans and non-citizens which composed 15% of the records. The third category came from animals, birds, insects, and worms which represented 7%. The fourth category was from commemorative events and meetings for ceremonies, incidences, rituals and religion representing 12% of the toponyms. The fifth category of toponyms arising from disasters such as diseases, landslides, lightning and earthquakes composed 1%. The sixth category came from household goods representing 5% of the toponyms. The seventh and last category was from toponyms of foreign origin including opaque (whose origin or meaning is unknown) ones which represented 3% of total toponyms in the gazetteer*.*

### Research questions sampling procedure, and sample size

The questionnaire aimed to answer three questions; (1) are there concurrent views of information regarding the historical heritage information on Kenya's geographical names among all dialects? (2) Are there places that share the same place name of a similar or divergent meaning? And (3) What are the best approaches to updating heritage data in a gazetteer service using public data and VGI data?

The next part of the research examines heritage meanings. Its compilation provided supplementary data to the characterized features and their toponyms based on 1567 toponyms extracted from a list of 26,600 place names found in official gazetteer records. A minimum of 390 responses was required (Yamane, 1967) for infinite populations at a confidence interval of 5% for the matched toponyms data of AGI and VGI compiled as of March 2020. As per the Yamane approach, the minimum sample size *n*, required by *n* = *N*/(1 + *N*(e^2^), where N represents the population size, and ‘e’ represents the error margin. At a 95% confidence level with a sample of 15,000 matching records, by substituting the values given, *n* = 15,000/ (1 + 15,000(0.05)^2^ shows 390 as the minimum required responses. However, to reduce bias and increase data coverage in this study, 438 respondents participated. This was way above the minimum required sample.

### Area of study

**Fig. 1:**
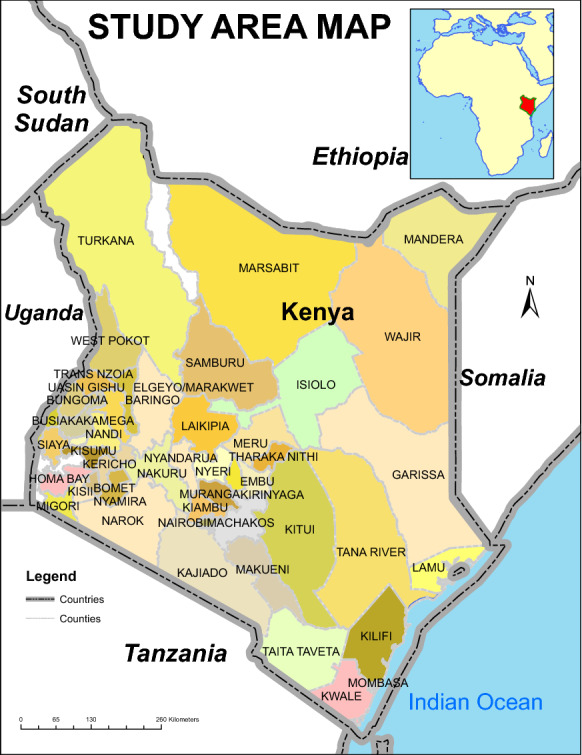
Area of study map

The research area was Kenya in East Africa. Kenya is located in—0.11 and 36.76 degrees of latitude and longitude respectively for the WGS84 coordinate system as shown in Fig. 1.

### Methods of assessing heritage on changing geographical names

**Fig. 2:**
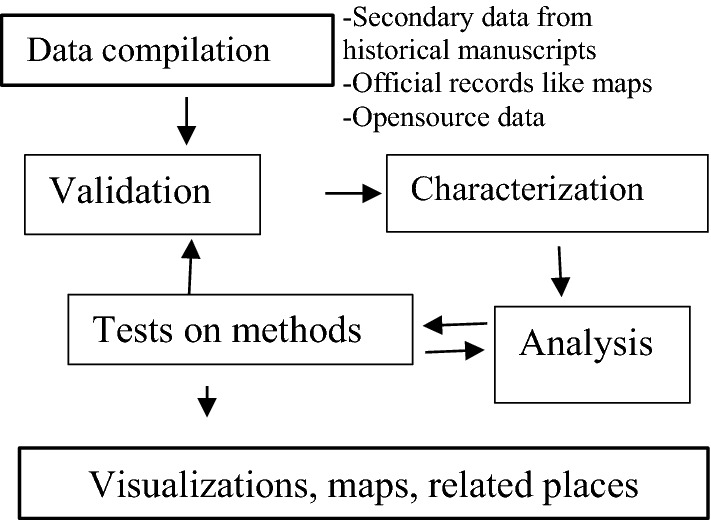
Methodology diagram

As shown in Fig. 2, the research compiled historical and official records to build a list for analysis in addition to data obtained from questionnaires.

The approach used to establish the sources of the geographical names involved historical archival records and personal interviews. The research used official maps and questionnaires as one of the methods to reduce bias (Blumer, 1954). The official records validated results such as supplementary material used to verify questionnaires’ data on toponyms by professions conversant with place names. The research on toponyms meanings used a questionnaire with open and close-ended questions.

Secondary data sources inferred include historical, VGI and official records. There was an application of a Google form questionnaire containing open and close-ended questions. We carefully selected the respondents. Most of the respondents were from the land sector that use toponyms in their transactions. The respondents were to declare whether they are natives to the places they described.

The Haversine formula(Inman, 1835) was used in validating the distance computation using longitude and latitude. In the direct projection of all coordinate distances of the associated related areas identified by respondents showed that the results concurred.

#### Data sources

**Fig. 3:**
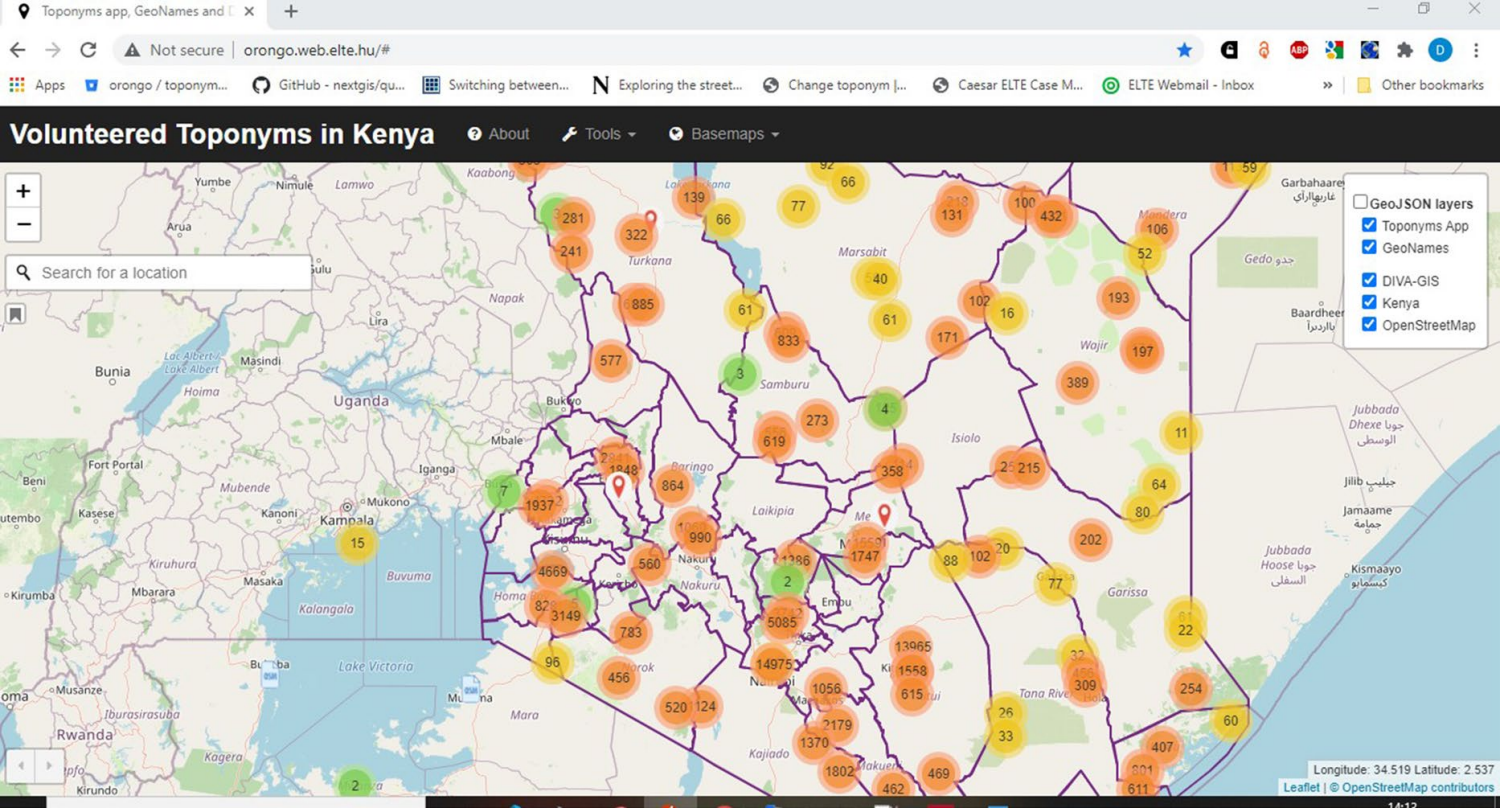
Visualization of VGI toponym data sources (https://orongo.web.elte.hu)

The study data include toponyms, administrative locations, meanings and dialects information obtained from secondary sources in addition to questionnaire responses from surveys conducted from April 2020 to October 2020. As at March 2020, the study considered VGI data from sources such as 31,172 data attributes from GeoNames, data from OSM (base map tile) and 26,600 DIVA-GIS records as shown in Fig. 3 and in Table 1 (available online at https://orongo.web.elte.hu) based on BootLeaf (Lead, 2018) to aid analysis. Before analysis, all VGI data was hooked into the Postgres database (Nyangweso & Gede, 2019). Authoritative public data of topographical maps and official archive records was also sourced to validate the results and take care of missed areas.

The research considered only 1567 toponyms that had an exact meaning traced from the dialects both in the official and VGI records. The toponyms were then grouped into seven categories based on 26,600 toponyms contained in the gazetteer. These toponyms were a compilation from questionnaire response data, historical records and published articles before being verified and validated by selected professionals to ensure that what is described by the toponyms is in the native speaking or locals’ common knowledge. The 26,600 toponym-features included administration boundary, hydrographic features, protected and sport features, populated, built-up and constructed structures, transport, topography and vegetation. Variables used to characterize include features availability and current usage, dialects and feature class of each toponym. Table 3 indicates the various data sources used in the research.

**Table 3:** Data sources as of March 2020

	Data type	Provider	Source
1	GeoNames data;.csv	GeoNames	http://www.geonames.org/datasources
2	Prototype gazetteer data;.csv	United States Board on Geographical Names	1962 Gazetteer data
3	AGI(official data),.csv	Kenya Open Data portal	http://www.opendata.go.ke/
4	OSM; web base map tiles	OSM(OpenStreetMap)	https://www.geofabrik.de/data/
5	Secondary historical data	Manuscripts,archives	KNBS (2019)
6	Question survey data	Toponyms questionnaire	http://mercator.elte.hu/~kdncx6/toponyms.xlsx

##### Related places in authoritative data

**Fig. 4:**
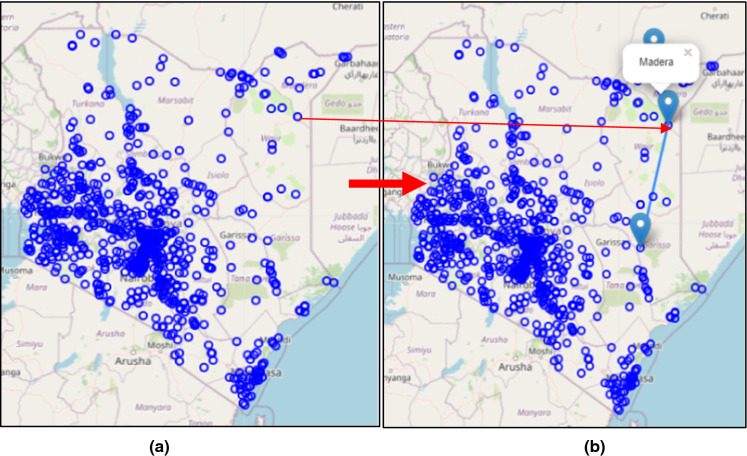
**a** Related places based on AGI, **b** Related places based on AGI. [Source: http://mercator.elte.hu/~kdncx6/relationss.html]

A total of 284 related places were extracted from authoritative data to cover the areas not covered by the respondents’ data. The data was compiled by excluding all those places mentioned by responses from the online questionnaire. The data was also prepared in a leaflet web map as shown in Fig. 4a and b. When visualizing the point data, move the computer mouse over the web map-point and then double-click on each of the points of interest to trigger the display window which identifies related places as illustrated in Fig. 4b.

**Fig. 5:**
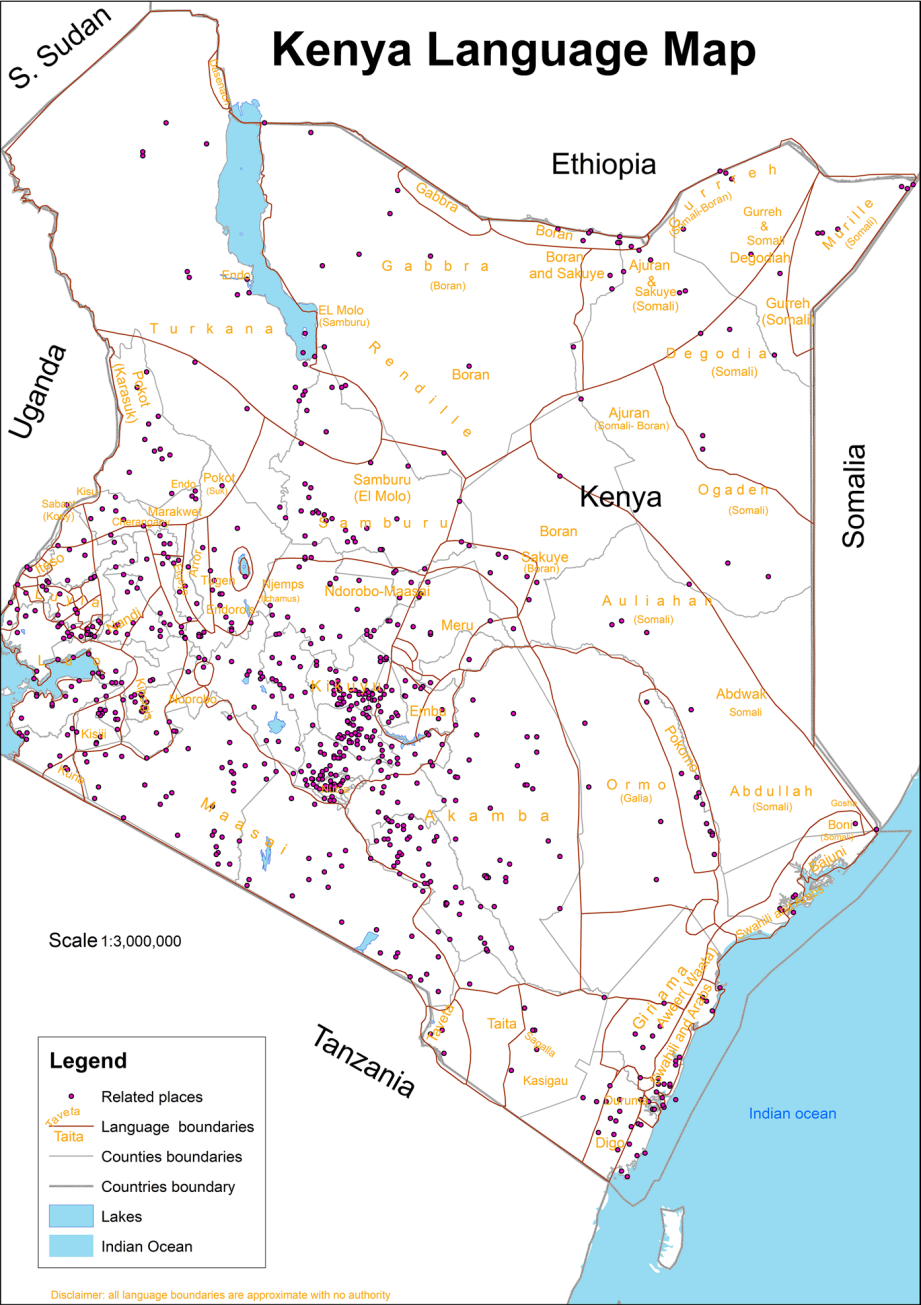
Language map of Kenya with 284 uniquely related places extracted from AGI records

There was insufficient data received from respondents from minority dialects such as the Mbeere, the Suba, the Rendille, the Bajuni, the Kenyan Asians, the Burji, the Ilchamus (Njemps), the Dorobo, the Walwana (Malakote), the Aweer (Waata), the Dasenach, the Makonde, the Wayyu, the Konso, the El Molo, the Gosha, the Kenyan Americans and the Dahalo. However, the insufficiency of the minority dialects’ data did not affect the outcome of the results. Authoritative data overlay and comparison made the analysis complete. The dialect boundary map as shown in Fig. 5 has 284 related places shown as points on the map. The boundary map was developed using VGI data after overlay with authoritative records. Visualization of the points is available online at http://mercator.elte.hu/~kdncx6/relationss.html.

##### Language map of Kenya

Figure 5 shows Kenya's language map containing an overlay of 284 related places identified in the authoritative data using Leaflet web map.

### Data processing and analysis

The applications used in processing and generating a visualized web map were Microsoft document applications, ArcMap Arcgis, SPSS, JavaScript, Python and HTML for spatial data entry. A semi-qualitative questionnaire was used to assess the information’s accuracy on the historical secondary data acquired from historical or secondary sources in order to verify the secondary data-sources. The questionnaire aim was to measure the user’s perceptions of place names in local-usages and their links on historical information as characterized in Table 2. The questionnaire also tackled issues of place names in terms of local usages for consideration in the prototype gazetteer service design. Respondents from most dialects participated in a questionnaire where twenty-five dialects participated. The respondents verified the compiled historical or written ethnographic reports. Some respondents identified toponyms from plant vegetation, animals and topography as they associated the toponyms’ local knowledge with official historical records especially the place names not mentioned in the gazetteer. The study used a selective or purposeful sampling method using Google forms to collect data and validate historical secondary data on current usage. Diverse data and access to information from different locations using Facebook messenger, WhatsApp and email inspired the primary data collection method because of sharing with a broad audience. This approach increased the response rate of the VGI data during the covid-19 pandemic when social distancing measures were enacted which curbed physical in-person data collection. In addition, the approach enabled the selection of the respondents to get the maximum amount of information regarding local knowledge and history for Kenya. Some respondents got involved in the processes of data collection where they were required to use map names while interpreting the associated heritage. From the questionnaire administration, 438 respondents in the study identified related toponyms within Kenya.

The respondents represented a majority count in Kenya by being residents or locals of an area or Kenyans. 22 dialects participated as shown in Table 4. There were no participants from the “other” category. Table 4 shows that out of the 438 respondents, 80% were male and 20% were female. The distributions of the respondent’s age group were such that 26–35 years represented 35.2% (154), 36-45 years composed 27.6% (120), 15–25 years represented 27.4% (120), 46–55 years represented 7.1% (31), those over 65 years represented 2.3% (10) and the least represented age group were those between 56 and 65 years at 0.5% (2). Regarding whether the responder knew what gazetteers are, 65.4% (282) of the respondents responded that they knew while 35.6% (156) of the respondents indicated that they did not know what gazetteers are. A total of 26 dialects that participated (see Table 4) identified about 497 related toponyms with about 235 toponyms being unique. Some respondents gave more than one related toponym and their history but only one response was responsive statistically. In order to assess the accuracy of the place name history provided, data-curation considered whether the responder was a native speaker in any related identified place(s). Being a professional native speaker helped make a possible choice of the various heritage data provided by the 438 respondents. Most professional respondents came from the land survey, geomatics, cartography, geodesy, information technology, engineering, law, farming, business and teaching disciplines. The research also found out that some toponyms came through mispronunciation mistakes before assuming permanent usages such as “Kariokor” for Carrier Corps, “Karantini” and “Karatina” for quarantine. Besides, some toponyms bear dialect names for populated areas or administrative units such as Meru, Embu, Kisii, Kikuyu, Samburu, Tharaka Nithi, Elgeyo Marakwet, Maragoli and Turkana as indicated in the compiled file of characterization data of toponyms. The stakeholder survey results in Table 4 did not fully collect data from all dialect groups due to a lack of access to respondents from sparsely inhabited areas who could provide response data of toponym source meanings from their location due to nomadic communities in the areas. The missing data may indicate that more research is needed to fully cover the non-documented sources of the sparsely populated areas which may involve the location of the respondents in their temporal shelters to get information.

**Table 4:** Questionnaire responses [April–October; 2020, number of responses = 438]

	Dialect name	No. of respondents	% of respondents		Dialect name	No. of respondents	% of dialects
1	Kalenjin	103	23.5%	13	Turkana	3	0.7%
2	Kisii	87	19.9%	14	Swahili	2	0.5%
3	Kikuyu	72	16.4%	15	Ilchamus	1	0.2%
4	Luo	57	13.0%	16	Mbeere	1	0.2%
5	Luhyia	24	5.5%	17	Pokomo	1	0.2%
6	Kamba	21	4.8%	18	Orma	1	0.2%
7	Maasai	19	4.3%	19	Sakuye	1	0.2%
8	Meru	15	3.4%	20	Samburu		
9	Embu	9	2.1%	21	Suba	1	0.2%
10	Nubi	8	1.8%	22	Kenyan European	1	0.2%
11	Mijikenda	6	1.4%	23	Others*	0	0
12	Taita	4	0.9%				
			Total			438	100%

## Results or findings

Historical information and the relation of the named places were core to the research. A total of 438 related place names and their associated histories were linked to associated related toponyms as shown in Fig. 2. Based on this, 235(53.7%) respondents gave unique related toponyms with related history. In comparison, 203 (46.3%) respondents presented repetitive place names, 238 (54.3%) responded to places as native speakers while 200 (45.7%) respondents related places that were not from their native language. The meaning of each toponym helped in classification and characterization based on explanations provided by the respondents. Two or more related toponyms but with precisely the same meaning were synonymous. Those toponyms similar in spelling and variation in meaning, were categorized as homonymous. Those with the same meaning but with differences in meaning depending on usage context belong to the hypernymy category. In curating the related toponyms, 360 (82.2%) toponyms provided were synonymous with each other, 41 (9.4%) historically associated, 32 (7.3%) homonymous and 2 (0.5%) opaque. Other toponyms hailed from hyperonymy, hyponymy and opaque categories with one toponym each based on a classification adopted in arborescent relations of toponyms (Bensalem & Kholladi, 2010).

**Fig. 6:**
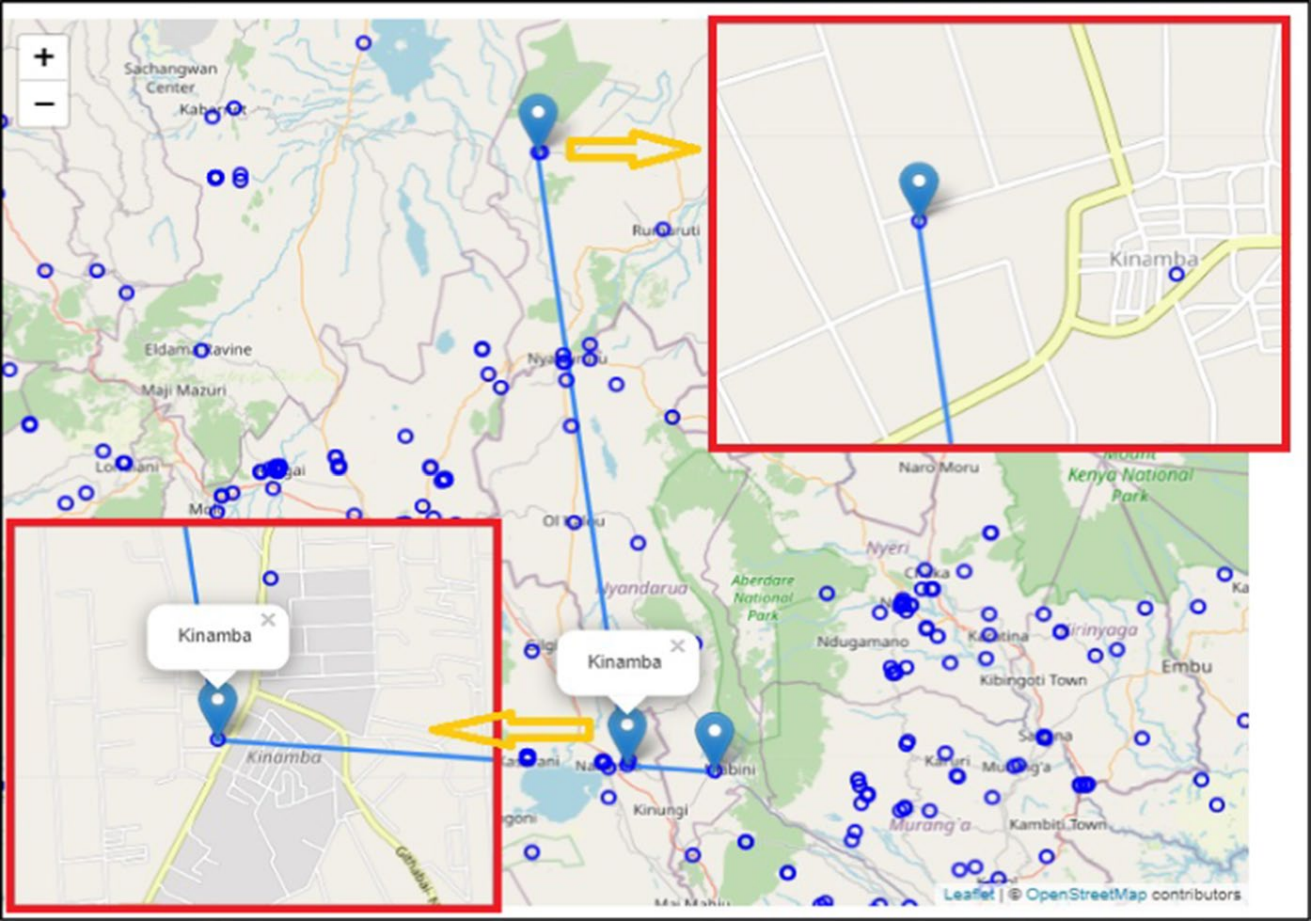
Map with related places (*n* = 438, unique place 235), Source: http://mercator.elte.hu/~kdncx6/relations.html)

Based on the respondents’ results, related places are constrained to the Western region and parts of Central Kenya as shown in Figs. 4, 5 and 6. Each point has related places with a similar name located elsewhere as indicated by the blue circles. These closely spaced circles indicate densely populated places where many names exist related to or used in other places for memory and identity. Besides, these are areas where dominant communities reside. The rest of the places are either scarce or have no related place. This places probably could be partially named due to sparse settlements, nomadic communities and lack of related places. In the scarcely populated areas, probably the place names are not currently mapped due to access and logistics needed. The distance separating the related places varied from less than 1 km for most places to 1087 km. For example, “Lomule” which is found in South Sudan and another “Lomule” or “Nomule” found in Gala Halima-Ethiopia. This example is associated probably with the Nubian settlements for colonial soldiers who were resettled in Kibera, Kenya. The names associated with the Nubian dialect include Lomule, Sarang'ombe, Toi and Manyani. The mean distance of separation for 235 unique related places was 147.6 km. The number of times respondents mentioned a related place varied with the respective number of usages. As indicated in Table 5, Majengo is mentioned 34 times, Rongai 25 times, Milimani 25 times, Makutano 16 times, Kasarani 12 times, Kisumu Ndogo 11 times, Kayole 10 times, Huruma 10 times and Bahati 9 times. The other places were mentioned less than thrice and either appeared used in one or two more places.

**Table 5:** 235 Related places (*n* = 438)

Toponym	No. of different locations identified
1.Majengo	9
2.Milimani	8
3.Makutano	7
4.Kisumu Ndogo	6
5.Matunda	5
6.Kaloleni	4
7.Karima	4
8.Soko Mjinga	4
9.Makongeni	3
10.Gem	3
11.Likoni	3
12.Kenyatta	3
13.Mulango	3
14.Moi	3

Toponyms such as *Baraton, Borabu, Bureti, Chepilat, Dandora, Gatundu, Kabianga, Kagumo, Kasarani,Kiganjo, Kigumo, Kinamba, Koguta, Kieni, Luanda, Mbitini, Mosiro, Muthaiga, Olasiti, Pipeline,Sabatia, Sengera* and *UasinGishu* appear in at least two different locations. All toponyms apart from the 14 indicated in Table 5 and the other 38 related toponyms appear in at least one other place thus indicating shared heritages.

The results also revealed that 173 (39.5%) respondents indicated that toponyms were named after topographical sources while 114 (26%) respondents stated that toponyms were named after persons and clans. Toponyms from foreign and unknown sources accounted for 98 (22.4%) respondents. 18 (4.1%) respondents linked toponyms to household items and 17 (3.9%) respondents responded that toponyms were named from animals and events (animals and events had 17 (3.9%) respondents each). Finally, the toponyms named after disasters was the least with 0.2% of the respondents. Results from the historical characterization of names based on seven categories were as shown in Table 2.

The Pearson correlation investigated the relationship between source dialects used in naming places and relationship types of toponyms. The results are as shown in Table 6. The scores assigned ranged from 1 to 7 namely synonymy, homonymy, hyperonymy, hyponymy, association, mistake and opaque. There was no significant relationship found between the same place name usages among dialects and distance. Besides, when correlating same place name usage among dialects and the relation type, the relation was positive, moderate in strength and statistically significant* r* (438), = 0.166, *p* < 0.001.

**Table 6:** Pearson's correlation (2 tailed *n* = 438)

	Correlations	No. of dialects	Relation type
Source dialects	Pearson corr	1	0.166
	Sig.(2-tailed)		0.000
	*N*	438	438
Relation type	Pearson corr	0.166	1
	Sig.(2-tailed)	0.000	
	*N*	438	438

The source dialect has a mean of 2.02 with a standard deviation of 0.172 while that of relation type has a mean of 1.50 with a standard deviation of 1.247 as shown in Table 7. This standard deviation values indicate that data was more spread out in relation types than in source dialects. Furthermore, dialect source data had consistent scores than in relation types.

**Table 7:** Means and standard deviation of data

Descriptive statistics	Mean	Std. deviation	*N*
Source dialect	2.02	0.172	438
Relation type	1.50	1.247	438

Concerning the issue of agreeing and knowing whether toponyms have historical information, 192 (43.8%) respondents strongly agreed, 212 (48.4%) respondents agreed, 33 (7.5%) respondents were neutral while 1 (0.2%) respondent disagreed. Based on the issues affecting Kenya's toponyms, 263 (60%) respondents concurred that changing toponyms was the most prevalent issue while 225 (51.4%) respondents identified using similar place names as an issue. In addition, the issues of adopted names, incomparable place names, conflicting place names, new place names and displaced places names were pointed out by 220 (50.2%), 155 (35.4%), 142 (32.4%), 105 (24%) and 75 (17.1%) respondents respectively. Most respondents were dissatisfied with field removal thus indicating that the data available should be processed as the guide in data collection. The residents preferred use of existing related toponyms. Regarding the issues such as displaced toponyms, new toponyms, conflicting toponyms, incomparable toponyms, adopted toponyms, similar and changing toponyms, 363 (82.9%), 333 (76%), 296 (67.6%), 283 (64.6%), 218 (49.8%), 213 (48.6%) and 175 (40%) respondents respectively accounted for those who negated. This suggest that changes in the existing place names cannot succeed easily. Coincidentally, there was a reported incidence in the Kenyan local daily regarding place name change such as Isiolo^2^ and Machakos^3^ due to political pressure demanding changes on the mentioned geographical names.

Platforms of updating gazetteer, mobile application and web gazetteer service enable local respondents to document each toponym's location and historical information. 14.4% (63) of the respondents indicated that use of the toponyms mobile app was very easy, 45.7% (200) said that it was easy to use, 31.7% (28) of the respondents marked it as slightly hard to use and 1.8% (8) of the respondents said it was hard to use. Regarding the use of the web gazetteer service, 14.6% (64) of the respondents said that it is very easy to use, 44.1% (193) marked it easy to use, 31.7% (139) ranked it moderate to use, 5.9% (26) of the respondents said it is slightly hard to use and 3.7% (16) said it is hard to use.

Based on choosing which platform to use in updating a gazetteer, 34.9% (153) of the respondents preferred to use a mobile app to collect data. In comparison, 10.5% (46) preferred to use a mobile to a web link for the gazetteer and 10.7% (47) chose to use a desktop computer. Further, 13% (57) of the respondents preferred to use both mobile app and desktop computer while 30.8% (135) said they would use all the devices suited for them.

Assessment on the use of Geographic Information System (GIS) data among the respondents was done. 75.1% (329) of the respondents said that they had used GIS data while 16.7% (73) said that they are not sure whether they have used GIS data and 6.6% (29) of the respondents indicated having not used GIS data. The results of those respondents who confirmed to have used GIS data concurs with those who confirmed to have used GIS data to derive other products and those who may have used a GIS web map.

The majority, 87.2% (382) of the respondents, indicated to have used free community-created maps like OpenStreetMap or Google maps, 5.3% (23) said that they are not sure whether they have used the web maps while 7.5% (33) of the respondents said that they have not used the web maps. On the use of community map, 285 (65.1%) respondents used it majorly for searching of a place name while the use in mapping and navigation, overlaying it with other data sets, geolocation in apps and websites and for other uses accounted for 209 (47.7%), 131 (29.9%), 109 (24.9%) and 2 (0.2%) respondents respectively. Using the map for other purposes and geolocation accounted for 412 (94.1%) and 329 (75.1%) respondents respectively. On the contrary, 307 (70.1%) respondents, 229 (52.3% respondents and 153 (34.9%) respondents negated the use of community map in overlaying data layers, mapping and navigation and searching for a place name respectively.

There was an assessment of the likely attribute-field to exclude gazetteer-card records when collecting card data for gazetteers. The results indicated that the majority which was 142 (32.4%) respondents suggested “others” as the first field to remove while remarks accounted for 61(13.9%) respondents as indicated in Table 8.

**Table 8:** Attribute fields data collection form for the gazetteer

Field removal proposal	Yes (Number and %)	No (Number and %)
Others	142 (32.4%)	296 (67.6%)
Remarks	61 (13.9%)	377 (86.1%)
Alternate name	50 (11.4%)	388 (88.6%)
Vernacular spelling	50 (11.4%)	388 (88.6%)
Approval notes	49 (11.2%)	389 (88.8%)
Derivation	46 (10.5%)	392 (89.5%)
International Phonetic address (IPA)	39 (8.9%)	399 (91.1%)
Sheet number	35 (8.0%)	403 (92.0%)
File reference	32 (7.3%)	406 (92.7%)
Feature class/feature code	29 (6.6%)	409 (93.4%)
Cartographic name	24 (5.5%)	414 (94.5%)
Grid reference	22 (5.0%)	416 (95.0%)
Original spelling	19 (4.3%)	419 (95.7%)
Map name	17 (3.9%)	421 (96.1%)
Historical association	16 (3.7%)	422 (96.3%)
Description	13 (3.0%)	425 (97.0%)
Location coordinates	13 (3.0%)	425 (97.0%)
Topographic feature	9 (2.1%)	429 (97.9%)

Table 8 shows the attribute-fields used in collecting toponym data such as alternate names, vernacular spelling, approval notes, derivation, International Phonetic Alphabet (IPA), sheet number, file reference, feature class/feature code, cartographic name, grid reference, original spelling, map name, historical association, location coordinates, description and topographic attribute-fields. Responses on these accounted for 50(11.4%), 50(11.4%), 49(11.2%), 46(10.5%), 39(8.9%), 35(8%), 32(7.3%), 29(6.6%), 24(5.5%), 22(5%), 19(4.3%), 17(3.9%), 16(3.7%), 13(3%) and 9(2.1%) for the attribute-fields respectively. Attribute- fields of topographic feature and location coordinates accounted for 429(97.9%) and 425(97%) of the responses respectively. 425(97%), 422(96.3%) 421(96.1%), 419(95.7%), 416(95%), 414(94.5%), 409(93.4%) and 406(92.7%) of the respondents disapproved removal of description, historical association, map name, original spelling, grid reference, cartographic name, feature class/feature code and file reference respectively as attribute fields in the gazetteer. Further, 403(92%), 399(91.1%), 392 (89.5%), 389(88.8%), 388(88.6%),388(88.6%), 377(86.1%) and 296(67.6%) negated the exclusion of the attribute fields of sheet number, International Phonetic Alphabet (IPA), derivation, approval notes, alternate name, vernacular spelling, remarks and others respectively from the gazetteer card record. I addition, most people considered that each of the gazetteer fields is critical to the richness of any database's detail. However, two attribute fields of ‘others’, and ‘remarks’ had 32% (142) and 13% (61) respectively of the respondents still responding that the fields be obliterated from gazetteer card record entries. The results after assessing attributes fields in gazetteer data collection forms indicate that the classical fields are still useful in maintaining data consistency on topographic maps.

## Discussion

Our method of classifying the sources of toponyms concurred with Stewart’s (1954) treatise, except that some classes were left out due to existing spatial heterogeneity for the area of study, limited available toponym data and synonymous reference terms of the classes in the study area. The respondents' description information was clear enough to distinguish meanings and their relationship with others located elsewhere. Some toponyms may have arisen by transfer from another language for use as if they originated from that language such as *Bomet* in *Kipsigis* dialect and *Bomani* in *Mijikenda* dialect from the name ‘*Boma’,* traditionally in Swahili dialect.

The use of authoritative and VGI data has a time attribute that indicates the history of most known and related places and clustered around significant towns. Simultaneously, the remote areas’ data was scanty, (Mahabir et al., 2017) as indicated by road datasets in Nairobi. VGI data coverage decreased as one moved away from urban centres to remote areas. Hence, the use of VGI data sources should supplement data collection technologies as per the themes available and favourable for remote areas. The involvement of professions that transact in processes that require toponyms helped in cleaning and improving data quality. The research captures the application and usage of VGI data collection in the local and urban settlements which concurs with the FINTAN project research (GFDRR & World Bank, 2018). The advances in technology and access of smart devices with the internet as per the ICT reports and tools have increasing usage globally (International Telecommunication Union (ITU), 2018), hence motivating most people to access the web maps and GIS data.

Based on historical descriptions and observations, the toponyms, if related, reveal a dialect's historical location at a named place in a specific time due to historical or current usage of the toponyms (Press, 2018). Most respondents provided information on toponyms outside their dialect hence indicating that local habitation contributes to some information on toponyms' source language. The exact origin and relations of the named places in the folk tales and songs sometimes are renamed, do not exist, or might have shifted. These include toponyms such as "Sot" and "*Kaplong,*" whose origin can be traced to Marakwet and Baringo regions as indicated by respondents. Urbanization also influences changing toponyms (Buchecker & Frick, 2020). Legislating and political activism also contribute to changes on African country names such as Gold Coast to Ghana, Dahomey to Benin, South Rhodesia to Zimbabwe and Haute Volta to Burkina Faso which all faced decolonization strategies. There is a difference in local usage of the place names for identity while officially, different toponyms exist for administration (Rose-Redwood et al., 2010) thus demeaning the official names. Toponyms on the public topographic maps are older than the year 1900 as mapped by the Ordinance Survey Company. Some toponyms described by activities especially in the nomadic areas no longer exist on maps other than local usage.

Sources of the toponyms’ naming strategies differ based on topographical features, names of dialects or clans, animals, birds, historical events, disasters, household goods or even foreign toponyms with unknown meanings. People who currently live and belong to associated dialects can precisely describe toponyms and develop the relation between cities by their movement activities (Meijers & Peris, 2018). The links could reveal different spatial relations if people's dialects came outside the urban areas. There was an agreement in the meaning of some of the toponym's names even though they have different spellings as indicated by the related places of heritage. This study of toponym heritage concurs with the study which modelled wild animal distribution using toponyms (Tattoni, 2019). For example, there is a place named after a leopard in Kipsgis's Bomet County identified as “*Kapsinendet*”, similar in meaning to another in Kirinyaga called “*Kangaru”,* named after a leopard in the Kikuyu dialect and “Kyawangu”, also named after a leopard in the Kamba language. There is replication of toponyms of features resulting from trees, bushes, plants, lightning, animals and plants which can only look the same across different dialects if only their meanings are known or translated from the written language. Toponyms model an ecological niche indicating past occupation of certain species of animals or plants as indicated by the toponyms' characterizations of related places or heritage names.

The research methodology on shared heritage had a weakness since not all toponyms were scrutinized exhaustively for all the regions in Kenya especially those occupied by pastoral communities in the Northern and Eastern parts of Kenya, due to remoteness of the areas. Furthermore, the lack of respondent data from the scarcely populated areas can be a challenge and need a successful mitigation strategy. Access to devices that can utilize the tools to gather toponyms, training users and logistics can also be a challenge to get reliable respondents. More research is recommended for the areas not covered in order to attain more equality and inclusivity on the heritage data.

## Conclusion

Based on the results from the respondent data and formal records, there exists shared heritage as indicated by same place name usage in 284 related places as shown in Fig. 4 (based on authoritative data) and 235 related places (based on respondent data) as shown in Figs. 4 and 5 respectively. The names attribute the motivation behind using loan words or shifts for toponyms to continued significance of heritage and the repeated same place names usages in different places. Furthermore, the toponym meanings reveal rare information such as common ancestry of origins and the environment's topographical conditions prevalent in the past. Moreover, the information obtained serves as heritage which connects the features’ historical extent and spread, distribution of feature, phenomenon mapped or how similar features relate based on toponym relationships as indicated by the diverse seven categorized characterizations. There was a limitation on the number of respondents and the exclusion of toponyms originating from mistakes, nicknames, opaque names or newly named places. Besides, not all toponyms were considered in the print gazetteer records since only 17,266 GeoNames matched with those in AGI’s catalog while 9334 (35%) toponyms did not match. The mismatched records of GeoNames indicate that toponyms might have changed or the features described no longer exist due to the developments and expansion of some towns and cities.

The research also confirmed the effectiveness of collecting heritage data using a crowdsourcing approach due to its capability of getting diverse views on heritage from a wide population. Respondents provided commonly known name-origins due to similar meanings of toponyms provided for the same names and repeated place mentions as indicated by the questionnaire response data. This enabled getting concurrent views of information regarding the historical heritage information on Kenya’s geographical names from different dialects. The research enabled collection of 519 related places with precisely the same toponyms usage in more than one place; out of which 235 came from questionnaire responses while 284 was availed from historical data, published reports and articles. There was no significant relationship found between the same place name usages among dialects as indicated by a positive weak correlation *r* (438), = 0.166, *p* < 0.001 based on the effect of using the related places and the distance between related places. Lastly, based on the challenges encountered in sharing online or mobile-based tools for collecting and updating heritage data, the use of offline-based tools in areas with no internet access is recommended. Further, there is need for allocation of more time of more than a year in collecting as much data as possible in order to fill the shortcomings of the heritage data.

The reliability of VGI records require checks for completeness against archived historical and official documents. These checks include novel mobile applications that work offline such as the Android-based toponyms app that incorporate offline data creation. The usage of the mobile apps in gathering heritage information in rural areas will help rural communities to collect insights on place names and notify county administrators for development purposes hence help the National Mapping Agencies in updating the place names database.

## Data Availability

All data generated or analysed during the study are included in this article and its supplementary information files. Questionnaire data: http://mercator.elte.hu/~kdncx6/toponyms.sav. Characterization categories:http://mercator.elte.hu/~kdncx6/characterization.pdf. Toponyms meanings: http://mercator.elte.hu/~kdncx6/meanings.pdf. VGI data visualization website:http://orongo.web.elte.hu.

## Footnotes

1 http://kso2.lantmateriet.se/

2 https://www.standardmedia.co.ke/adblock?u=https://www.standardmedia.co.ke/rift-valley/article/2001305189/leaders-push-to-rename-airport

3 https://nairobinews.nation.co.ke/news/road-renamed-sonkos-father

## Code Availability

Code is available on request for toponyms app and web-maps.
